# Supplementary material for: Structural Insights Into the High Selectivity of the Anti-Diabetic Drug Mitiglinide
Source: Front Pharmacol. 2022 Jun 30;13:929684. doi: 10.3389/fphar.2022.929684 (PMC9279661; doi:10.3389/fphar.2022.929684)
Supplement: Supplementary file 1 [file Presentation1.pdf]

Supplementary Material

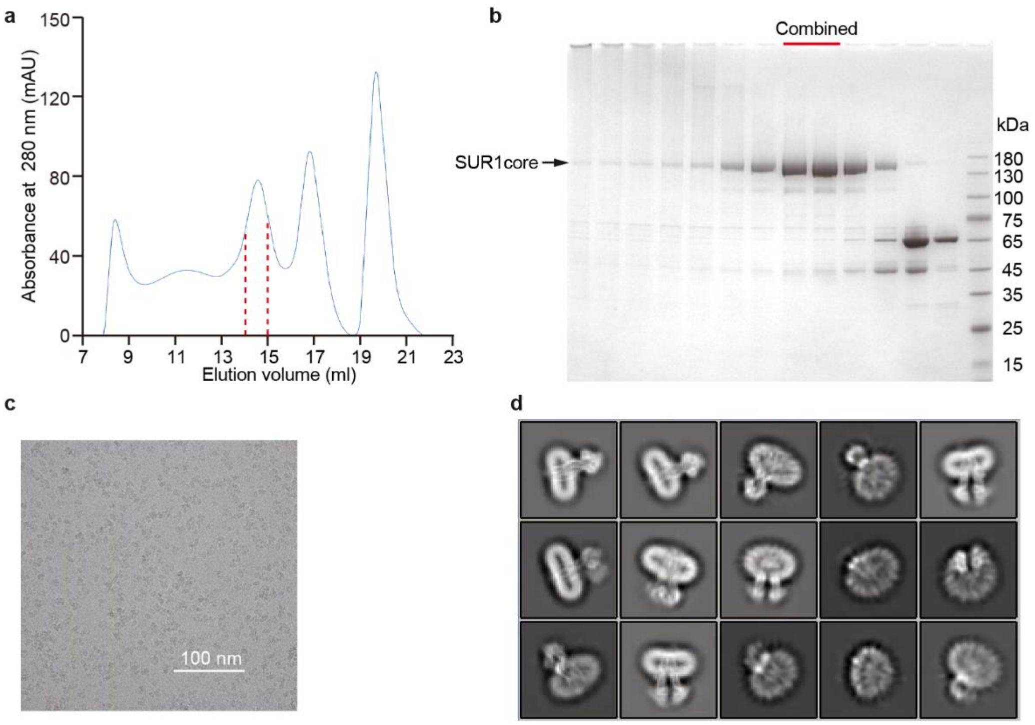

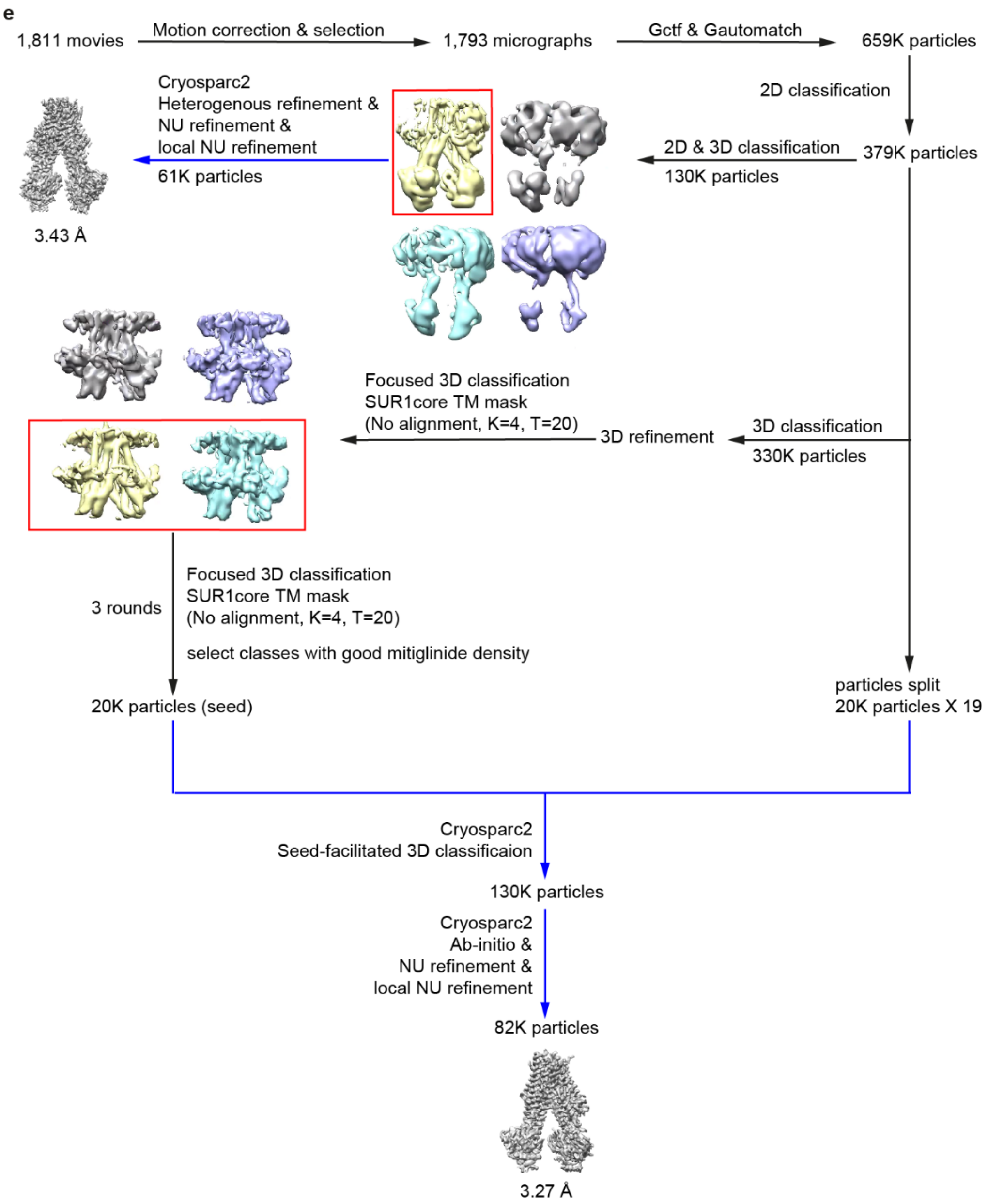

Supplementary Figure 1. Cryo-EM sample preparation and data processing flow chart. **a**, Size-exclusion chromatography (SEC) elution profile of SUR1core. Fractions between the dashed lines were used for cryo-EM sample preparation. **b**, SDS-PAGE detection of SUR1core. Fractions labeled by red bars were collected for cryo-EM sample preparation. **c**, Representative raw micrograph. **d**, Representative 2D class averages. **e**, Flow chart of cryo-EM image processing of SUR1core in complex with RPG.

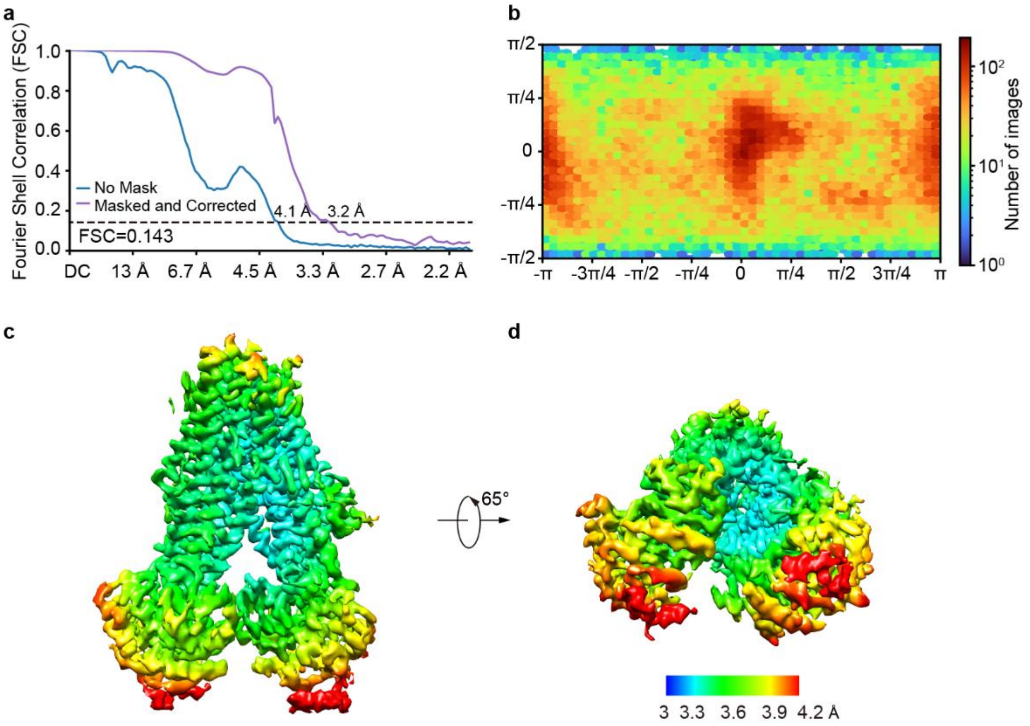

Supplementary Figure 2. Parameters for the final refined SUR1core Cryo-EM map. **a**, FSC curve of the final refined map is shown. **b**, Angular distribution of SUR1core particles for the reconstruction of the final refined map. **c-d**, Local resolution of the final refined map.

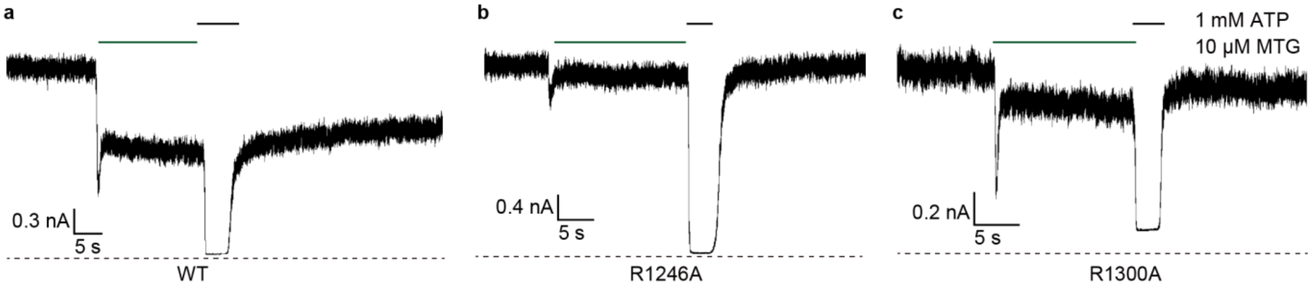

Supplementary Figure 3. Representative recordings of MTG inhibition of K_{ATP} channels. **a-c**, The inhibition of 10 μM MTG inhibits on the inside-out currents of Wild-type, R1246A and R1300A K_{ATP} channels.

Supplementary Table 1
Cryo-EM data collection, refinement and validation statistics

	SUR1core
PDB ID	7WIT
EMDB ID	EMD-32535
**Data collection and processing**	
Magnification	135,000 ×
Voltage (kV)	300
Electron exposure (e$^-$/Å^2)	50
Defocus range (μm)	-1.5 to -1.8
Pixel size (Å)	1.045
Symmetry imposed	*C1*
Initial particle images (no.)	659,663
Final particle images (no.)	82,717
Map resolution (Å)	3.27
FSC threshold	0.143
Map resolution range (Å)	3.27 - 250
**Refinement**	
Initial model used (PDB code)	6JB1
Model resolution (Å)	3.68
FSC threshold	0.5
Model resolution range (Å)	3.68 - 250
Map sharpening B factor (Å^2)	-117.8
Model composition	
Non-hydrogen atoms	8,109
Protein residues	1,133
Ligands	2
B factors (Å^2)	
Protein	60.47
Ligand	66.99
R.m.s. deviations	
Bond lengths (Å)	0.005
Bond angles (°)	0.544
Validation	
MolProbity score	1.84
Clashscore	5.03
Poor rotamers (%)	3.11
Ramachandran plot	
Favored (%)	96.77
Allowed (%)	3.23
Disallowed (%)	0.00
